# Factors Associated with Modern Contraceptive Use among Married Women Attending Comprehensive Health Centers (CHCs) in Kandahar, Afghanistan

**DOI:** 10.1155/2021/6688459

**Published:** 2021-04-06

**Authors:** Mirwais Saheem, Muhammad Haroon Stanikzai, Najeeb Rahimy, Najibullah Fazli, Ghulam Mohydin Mudasir, Hadia Sayam

**Affiliations:** ^1^Master of Public Health Program, Faculty of Medicine, Kandahar University, Kandahar, Afghanistan; ^2^Public Health Department, Faculty of Medicine, Kandahar University, Kandahar, Afghanistan; ^3^Para Clinic Department, Faculty of Medicine, Kandahar University, Kandahar, Afghanistan; ^4^Internal Medicine Department, Faculty of Medicine, Kandahar University, Kandahar, Afghanistan; ^5^Para-clinic Department, Malalay Institute of Higher Education, Kandahar, Afghanistan

## Abstract

**Background:**

Modern contraceptives are highly effective and reliable methods of preventing unintended pregnancies and reducing maternal deaths. Only 22 percent of currently married women use modern methods of contraceptives in Afghanistan. This study assessed the factors associated with modern contraceptive use among married women attending comprehensive health centers (CHCs) in Kandahar Province.

**Methods:**

This was an institution-based cross-sectional study that included 325 married women who attended randomly selected comprehensive health clinics in Kandahar between September and October 2019. The total sample size was allocated proportionally to selected health clinics based on the recent 3-month average patients load. We used a consecutive sampling method to select study participants. Data were collected in a structured questionnaire, which included information on respondents' demographic, socioeconomic, reproductive, and contraceptive experiences. Data was analyzed using SPSS 21.00 statistical software. We used descriptive statistics such as tables and proportions to present data. Binary and multiple logistic regression analyses were carried out to determine factors associated with modern contraceptive use.

**Results:**

Out of 325 married women, 127 used modern contraceptives with a prevalence of 39.1% (95%CI = 33.7%–44.6%). The results indicated that the area of residence (AOR = 2.61, 95% CI 1.43-4.78) and ever use of contraceptives (AOR = 14.92, 95% CI 6.88-32.34) are associated with modern contraceptive use among married women attending comprehensive health centers in Kandahar.

**Conclusion:**

This study found that modern contraceptive use was higher than reported on the national level. The most persistent factors associated with modern contraceptive use in this study were urban residence and ever use of contraceptives. As a policy measure, family planning programs should be prompted to the rural residency in Kandahar Province.

## 1. Background

The Maternal Mortality Ratio (MMR) in Afghan women continues to be a major public health concern and remains the highest in the region. It is estimated to be 638 deaths per 100000 live births in 2017. Afghanistan also has one of the world's highest fertility rates (4.5), and related to this is the high infant mortality rate. In Afghanistan, only 22 percent of currently married women used any contraceptive method in 2017 [1]. Contraceptive use encompasses potential benefits in terms of maternal health, economic developments, educational advances, and women empowerment. Therefore, it is considered one of the most successful interventions of the last 50 years.

The World Health Organization (WHO) defines contraceptives as a principal strategy that promote maternal and child health through adequate birth spacing and avoiding unintended pregnancies [2, 3]. Globally, it is estimated that 62 percent of married women or in union women of reproductive age (15-49) were using some form of contraceptive and 58 percent a modern method of contraceptive in 2017. The proportion of contraceptive use varies significantly by region ranging from a low 36 percent in Africa to a high 75 percent in North America [4].

Family planning is a vital component of comprehensive health care. Unintended pregnancies and inadequate birth spacing have significant adverse health effects. It can deteriorate women's health and raise neonates' risk of adverse health outcomes [5, 6]. WHO and other international organizations recommend 2-3 years of birth spacing to reduce child mortality and also improve maternal health [2]. However, recent studies have suggested that 3-5 years of birth spacing might be more advantageous [4–6].

Contraceptive use is affected by many factors such as socioeconomic factors including women' age, education, residency, and occupation [7–10]; reproductive factors including marital age, gestational age, gravidity, parity, and birth spacing [8–10]; and other factors including awareness regarding contraceptives, poor access to healthcare, access to media, and various disease-related issues [7–9, 11–15]. Understanding the factors influencing contraceptive use among those married women who are at risk for unintended pregnancies is essential for the development of effective family planning programs. Given Afghanistan's high fertility and MMR background, this study was aimed at determining the proportion of modern contraceptive use and elucidating factors that are associated with modern contraceptive use among married women who visit comprehensive health centers (CHCs) in Kandahar Province.

## 2. Methods

### 2.1. Study Setting and Period

This institution-based study was conducted in four out of thirteen randomly selected CHCs in Kandahar Province between September and October 2019. The data was collected from the outpatient departments of two rural (Sarkari Bagh and Mandisar) and two urban (Balakarz and Dr. Amirjan) CHCs. These comprehensive health clinics provide a wide range of primary healthcare services including general medical consultation, vaccination, and family planning services.

### 2.2. Study Design and Population

This was an institutional-based cross-sectional study recruiting consecutive married women attending CHCs for routine healthcare.

For inclusion and exclusion criteria, we included consenting married women attending selected CHCs for healthcare services. Pregnant women, women aged 15-49 who were not married, and those who were unable to respond due to serious illness or unwillingness to participate in the study were excluded.

### 2.3. Sample Size and Sampling Procedures

A total of 325 married women were included in the study and estimated by a single population proportion formula (*n* = *Z*^2^*P* (1 − *P*)/(*d*)^2^) with the assumptions of the proportion of modern contraceptive use in Kandahar (*P* = 28.6%) [1], 95% confidence interval, the margin of error 5%, design effect of 1.0, and 10% nonresponse rate.

With regard to the sampling methods, four (2 rural and 2 urban) out of thirteen health clinics were randomly included in the study. The possible number of study participants between health clinics was allocated proportionally based on the recent 3-month average patient load. We consecutively included consenting married women who visited outpatient departments of selected CHCs for healthcare services on all working days during the study period.

### 2.4. Data Collection Methods

We used a structured and pretested questionnaire to collect the required information. The questionnaire was first prepared in English; later, it was translated into the local language (Pashtu) by experts and back to English to ensure that meanings of translation were not lost during translation. Three female clinical nurses collected data after being trained by the principal investigators. During training, the questionnaire was pretested on 20 participants in a different setting (Kandahar medical faculty teaching hospital) with the aim of estimating the time required to complete the questionnaire and revising the poorly structured questions. It was estimated that each interview would take up to 10-15 minutes. The data collection process was closely monitored to ensure that questionnaires were correctly filled.

### 2.5. Variables

#### 2.5.1. Independent Variables

The variables were grouped into three categories: sociodemographic, socioeconomic, and reproductive variables.

#### 2.5.2. Dependent Variables

The outcome variable in this study was the current use of a modern contraceptive method by married woman or her partner.

### 2.6. Operational Definitions

#### 2.6.1. Contraceptive Use

“The percentage of women married aged 15 to 49 who are currently using, or whose husband is using, at least one modern method of contraception, regardless of the method used” [2, 3].

#### 2.6.2. Modern Methods of Contraception

They included “female and male sterilization, oral hormonal pills, the intra-uterine device (IUD), the male condom, injectables, the implant (including Norplant), vaginal barrier methods, the female condom and emergency contraception” [2, 3].

### 2.7. Data Analysis

The collected data were entered and coded using Microsoft Excel (2019) and analyzed using IBM SPSS version 21 [16]. We carried out descriptive statistics for frequencies. Both bivariate and multivariate analyses were carried out to find an association between dependent and independent variables. All variables with a *P* value < 0.25 in bivariate analysis were included in the multivariate analysis to control the confounding effects. The *P* value of <0.05 was considered statistically significant.

### 2.8. Ethical Consideration

The study was approved by the Ethical and Research committee, Faculty of Medicine, Kandahar University. Administrative approval was obtained from the Kandahar public health directorate. In addition, after explaining the purpose of the study, informed written consent was obtained from all study participants.

## 3. Results

### 3.1. Sociodemographic Characteristics of the Study Participants

A total of 325 married women were included in the analysis making a response rate of 93.3%. The mean age of the respondent was 28 (±7.1) with minimum and maximum values of 15 and 49, respectively. Of the total, 118 (36.3%) respondents were of the age range 20 to 25 years. With regard to educational status, 298 (91.7%) respondents and 261 (80.5%) of respondents' husbands had no formal education. Most of the respondents (309, 95.1%) did not have a formal occupation and were housewives. More than half (215, 66.2%) of households had more than 10000 (Afghani) monthly income.  Table 1 shows the sociodemographic information of study participants.

### 3.2. Reproductive Characteristics of the Study Participants

The mean age of respondents at marriage was 17.3 (±3.2). Overall, 165 (50.8%) participants out of 325 were married before the age of eighteen. Of the 310 respondents with children, 161 (51.1%) had one to four children. Of all the study participants, 250 (76.9%) and 271 (83.4%) had ANC and PNC visits, respectively. The detailed characteristics of the respondent's reproductive variables are shown in Table 2.

### 3.3. Knowledge and Practice Pattern of Contraceptives

The results regarding knowledge and practice patterns of the participants on contraceptives are presented in Table 3. Of the total, 307 (94.5%) stated that they had knowledge about contraceptives. About half (49.2%) of the study participants had known more than one method. Other common methods known were pills (76, 24.8%), injectables (55, 17.9%), condoms (13, 4.2%), and intrauterine devices (IUD) (12, 3.9%). The main sources of information on contraceptives were health facilities (188, 61.2%) and peers (68, 22.1%). Other sources of information were parents (45, 14.7) and media (6, 2%).

**Figure 1:**
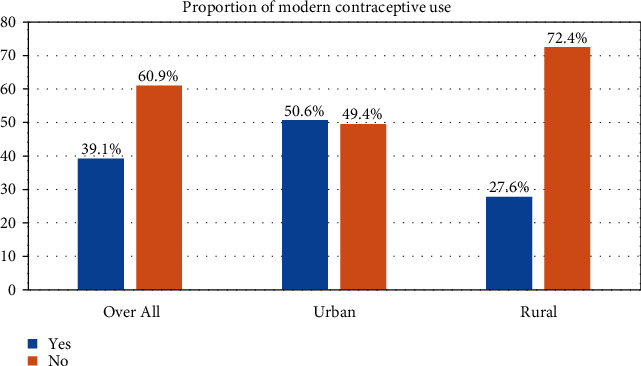
Proportion of contraceptive use among married women attending comprehensive health clinics in Kandahar Province, Afghanistan, 2019.

Of all the study participants, 127 (39.1%, 95%CI = 33.7%–44.6%) had currently used at least one of the modern contraceptive methods, varying from 50.6% to 27.6% in urban and rural settings, respectively (Figure 1).

Concerning the type of method used, 45 (34.9%) were on pills, 36 (28.6%) were on injectables, 26 (20.6%) were on condoms, and 19 (15.1%) were using IUD. The majority (72.6%) of the participants acquired their contraceptives from public health facilities. With regard to the ever use of contraceptives, 176 (54.2%) participants reported that they had at least used one of the contraceptive methods. Major reasons for using contraceptives were child spacing (78.5%) and prevention of unintended pregnancies (19.2%). Of the 176 ever contraceptive users, 114 (64.5%) participants have experienced side effects. Among 149 nonusers, common reasons for not using contraceptives stated were religious prohibition (51, 34.2%), wanting to have more children (37, 20.8), and fear of side effects (25, 16.8%).

### 3.4. Factors Associated with Modern Contraceptive Use

We conducted the bivariate analysis for all independent variables. Multiple logistic regression analysis was done for those variables with a *P* value of <0.25 in bivariate analysis. The results of multiple logistic regression showed that married women from the urban residence had 2.61 times higher odds of currently using contraceptives than the odds of married women with rural residence (AOR = 2.61, 95% CI:1.43-4.78). This study also found that married women with previous contraceptive use had 14.92-fold higher odds of currently using contraceptives compared to those who had not used contraceptives in the past.  Table 4 summarizes the results of bivariate and multivariable analyses.

## 4. Discussion

This cross-sectional study investigated the factors associated with modern contraceptive use among married women attending comprehensive health clinics in Kandahar Province. Understanding the factors influencing contraceptive use is of particular importance in countries like Afghanistan that have been affected by high MMR and infant mortality rate (IMR). This is a unique study providing a picture of both urban and rural populations. The findings revealed that the prevalence of modern contraceptive use was 39.1%, varying from 50.6% to 27.6% in urban and rural settings, respectively. This finding was higher than reported on the national level [1] and other studies conducted in Afghanistan [15, 17, 18]. This overestimation can be explained by the fact that married women visiting health clinics for seeking healthcare are more likely to use contraceptives than their counterparts. This may be the case as these health facilities are rendering family planning services. In this study, modern contraceptive use was lower than that of other studies conducted in India (52.2%) [9], Pakistan (49.7%) [10], Iran (72.9%) [11], and the United Arab Emirates (61.8%) [12]. This variation in contraceptive use reported in studies could be attributed to variation in cultural attitudes towards contraceptive use, access and availability of contraceptives in the study area, socioeconomic status, research design, and other nonexplored factors.

In this study, the main methods of modern contraception are oral contraceptive pills (34.9%) and injectables (28.6%), which is inconsistent with a prior study conducted in Afghanistan [15]. Major reasons cited for using modern contraceptives were child spacing and women's desire to prevent unintended pregnancies. These findings were consistent with the study conducted in Afghanistan [15] and similar studies conducted in Ethiopia [7] and Ghana [8]. On the other hand, religious prohibitions and wanting to have more children were the common reasons for not using modern contraceptives. This finding is in line with studies from Bangladesh [13] and Ethiopia [14].

In our study, the common source of information and acquiring contraceptives by married women were health facilities. This finding is consistent with the national report [1] and with a recent research report conducted in Afghanistan [15]. Since educational level was not associated with contraceptive use, all women of reproductive age including educated ones should receive health education on family planning. It is also imperative that a massive public health program aimed at improving awareness on the family planning methods should be undertaken in the light of low health literacy among the general population in Kandahar Province.

This study revealed that women with urban residency were nearly three times more likely to use modern contraceptives than women from rural residency. This finding is similar to that of a study from Afghanistan [15] and other studies from Pakistan [4] and Bangladesh [13]. The possible reasons for high use in urban dwellings include better socioeconomic status of women and cultural disparity. Furthermore, limited access and availability of family planning services in the rural area might further limit the use of modern contraceptives. In the current study, women who ever used contraceptives were more likely to use contraceptives, which is also supported by studies conducted in Uganda [19], Pakistan [20], and Bangladesh [21].

Factors shown to be associated with modern contraceptive use in previous studies such as age, income, education, parity, and occupation of married women were not associated in this study [8, 9, 11–15]. This may indicate possible differences in age, income, education, parity, and occupation of study participants.

## 5. Limitations of the Study

This study found crucial factors for modern contraceptive use in married women. The findings of this study, however, should be considered in light of its limitations. Since this study was an institutional-based one, the proportion of modern contraceptive use might have been overestimated and may not reflect what is going on at the community level. The cross-sectional nature of the study limits the causality effects between variables.

## 6. Conclusion

In this study, the proportion of modern contraceptive use among married women was 39.1 percent, which suggests an increase from the past studies. Urban residency and ever use of contraceptives were the factors associated with modern contraceptive use. As a policy measure, family planning programs should be prompted to the rural residence in Kandahar Province. Improving availability and access in rural areas are highly recommended to enhance modern contraceptive use. The results also suggest the need for qualitative research to divulge more information on religious and cultural beliefs influencing modern contraceptive use.

## Figures and Tables

**Table 1:** Sociodemographic characteristics of married women attending comprehensive health centers in Kandahar, Afghanistan, 2019 (*n* = 325).

Variable	Frequency	Percentage (%)
Age (completed years)		
1. <20	24	7.4
2. 20-25	118	36.3
3. 26-34	117	36.0
4. ≥35	66	20.3
Residence		
1. Urban	162	49.8
2. Rural	163	50.2
Women educational status		
1. No formal education	298	91.7
2. Basic	15	4.6
3. Secondary	7	2.2
4. High school graduate	5	1.5
Husband level of education		
1. No formal education	261	80.5
2. Basic	25	7.5
3. Secondary	13	4.0
4. High school graduate	26	8.0
Women occupation		
1. Formally employed	16	4.9
2. Unemployed/housewife	309	95.1
Husband occupation		
1. Formally employed	190	58.5
2. Unemployed	135	41.5
Number of household members		
1. 1 to 4	54	16.6
2. 5 or more	271	83.4
Household monthly income in Afghanis		
1. 5000-10000	70	21.5
2. >10000	215	66.2
3. Do not disclose	40	12.3

**Table 2:** Reproductive variables of married women attending comprehensive health centers in Kandahar, Afghanistan, 2019 (*n* = 325).

Variable	Frequency	Percentage (%)
Age at marriage		
1. <18	165	50.8
2. ≥18	160	49.2
Years in marriage		
1. <5	74	22.8
2. ≥5	251	77.2
Number of living children		
1. No child	15	4.6
2. 1 to 4	166	51.1
3. 5 or more	144	44.3
Age at the 1st child		
1. <18	117	37.5
2. ≥18	193	62.5
Child mortality experience		
1. Yes	53	16.3
2. No	272	83.7
Antenatal care visits		
1. Yes	250	76.9
2. No	75	23.1
Postnatal care visits		
1. Yes	271	83.4
2. No	54	16.6

**Table 3:** Knowledge and practice patterns of contraceptives by respondents.

Variable	Frequency	Percentage (%)
Knowledge of contraceptives		
1. Yes	307	94.5
2. No	18	5.5
Source of information (*n* = 307)		
1. Health facility	188	61.2
2. Media	6	2.0
3. Parents (mother-in-law)	45	14.7
4. Peers	68	22.1
Contraceptive methods known (*n* = 307)		
1. Pills	76	24.8
2. Condoms	13	4.2
3. Injectable	55	17.9
4. IUD	12	3.9
5. More than one	151	49.2
Currently using contraceptives		
1. Yes	127	39.1
2. No	198	60.9
Type of method used (*n* = 127)		
1. Pills	45	34.9
2. Condoms	26	20.6
3. Injectable	36	28.6
4. IUD	19	15.1
5. More than one	1	0.8
Where do respondents acquire their contraceptives (*n* = 127)		
1. Public hospital	92	72.6
2. Pharmacy	35	27.4
Ever use of contraceptives		
1. Yes	176	54.2
2. No	149	45.8
Reasons for using contraceptives (*n* = 176)		
1. Prevents unintended pregnancies	34	19.2
2. Prevents STIs	2	1.1
3. Helps to space children	138	78.5
4. Prevention of anemia	2	1.1
Experienced any side effects (*n* = 176)		
1. Yes	114	64.5
2. No	62	35.5
Side effects experienced (*n* = 114)		
1. Vaginal bleeding	28	24.6
2. Abdominal pain	10	8.9
3. Headache	34	29.9
4. Weight gain	18	16.1
5. Hypertension	18	16.1
6. More than one	5	4.4
Reasons for not using contraceptives (*n* = 149)		
1. Against religion	51	34.2
2. Opposition from husbands	24	16.2
3. Fear of side effects	25	16.8
4. Want to have more children	37	20.8
5. No knowledge	18	12.0

**Table 4:** Factors associated with modern contraceptive use among married women attending comprehensive health centers in Kandahar Province, 2019 (crude and adjusted odds ratio).

Independent variable	Categories	Contraceptive use		Crude odds ratio (95% CI)	Adjusted odds ratio (95% CI)
		Yes	No		
Residency	Urban	82	80	2.68 (1.69-4.26)	2.61 (1.43-4.78)
	Rural	45	118	1	1
Husband education	Educated	38	26	2.82 (1.61-4.94)	—
	Uneducated	89	172	1	—
Husband occupation	Employed	87	103	2.00 (1.25-3.19)	—
	Unemployed	40	95	1	—
Household income	5000-10000	21	49	1	—
	>10000	98	117	1.95 (1.09-3.48)	—
Years in marriage	1 to 4	17	57	1	—
	5 or more	110	141	2.61 (1.44-4.74)	—
ANC visit	Yes	108	142	2.24 (1.25-3.99)	—
	No	19	56	1	—
Previous contraceptive use	Yes	113	63	17.29 (9.20-32.49)	14.92 (6.88-32.34)
	No	14	135	1	1

## Data Availability

The primary data used to support the findings of this study are available from the corresponding author upon request.

## Supplementary Materials

Supplementary Materials Data collection questionnaire.
