# Supplementary material for: Factors Associated with Modern Contraceptive Use among Married Women Attending Comprehensive Health Centers (CHCs) in Kandahar, Afghanistan
Source: Int J Reprod Med. 2021 Apr 6;2021:6688459. doi: 10.1155/2021/6688459 (PMC8046549; doi:10.1155/2021/6688459)
Supplement: Supplementary Materials — Data collection questionnaire. [file 6688459.f1.docx]

**Factors associated with modern contraceptive use among married women attending comprehensive health clinics in Kandahar Province**

**File No: …………………………**

**Health facility Name: ……………………………**

**Part A: Socio-demographic Information**

| Questions | Response |
| --- | --- |
| 1. Name |  |
| 1. Age in completed years |  |
| 1. Address |  |
| 1. Residency | 1. Rural 2. Urban |
| 1. Education | 1. Primary 2. Secondary 3. High school graduate 4. Masters/PhD 5. No formal education |
| 1. Husband Education | 1. Primary 2. Secondary 3. High school graduate 4. Masters/PhD 5. No formal Education |
| 1. Occupation | 1. Housewife 2. Employer Specify ………………………. 3. Jobless |
| 1. Occupation of Husband | 1. Employer Specify ……………………….. 2. Jobless 3. Don’t Know 4. Don’t Disclose |
| 1. Family Monthly income |  |
| 1. Number of family numbers including you |  |
| 1. Age at marriage |  |
| 1. Number of years in Marriage |  |
| 1. Number of children |  |
| 1. Age at first birth |  |
| 1. Age of last child |  |
| 1. Child mortality Experience | 1. Yes 2. No |
| 1. Have you attended ANC visits? | 1. Yes 2. No |
| 1. Number of ANC visits |  |
| 1. Have you attended PNC visits? | 1. Yes 2. No |

**Part B: Contraceptive Knowledge, Consumption & Demand and supply**

| Questions | Response |
| --- | --- |
| 1. Have you heard about family planning? | 1. Yes 2. No ; If No skip Q. No21, 22 |
| 21. From where did you  hear about FP? | 1. Health facility 2. Friend 3. Husband 4. Mother –in law 5. If others specify …………….. |
| 22. How many methods of  Family planning do you  know? | 1. Pills 2. Condom 3. Inject able 4. IUD 5. LAM 6. If others Specify ………… |
| 1. Have you ever used FP method? | 1. Yes 2. No ; If No skip Q No. 24, 25,26,27,28, 29,30,31 |
| 1. Merits of using contraceptive? | 1. Prevention of unwanted pregnancy 2. Spacing 3. Prevention of STDs 4. Prevention of anemia and abortion 5. If others Specify ………… |
| 1. Was your husband aware? | 1. Yes 2. No |
| 1. Which method did you use? | 1. Pills 2. Condom 3. Inject able 4. IUD 5. Lam 6. If others Specify ………… |
| 1. Decision making in contraceptives? | 1. Husband 2. Wife 3. Both 4. If others Specify ………… |
| 1. Have you experienced being notable to use contraceptives? | 1. Yes 2. No |
| 1. What was the reason? |  |
| 1. Have you noticed any side effects? | 1. Yes 2. No ; If No skip Q No.31 |
| 31.What are the side effects  You encountered? | 1. Vaginal Bleeding 2. Abdominal Pain 3. Headache 4. Weight gain 5. Hypertension 6. If others Specify ………… |
| 32.Are you a current FP  user? | 1. Yes 2. No if No skip Q. No 32 |
| 33. Which method do you  use? | 1. Pills 2. Condom 3. Inject able 4. IUD 5. LAM 6. If others Specify ………… |
| 34. Did you change FP  Method? | 1.Yes  2. No |

Study location: Dr.Amirjan,Balakerz,Sarkari bagh and Mandhisar CHCs

Date of Interview: ……………………………………………..

Name of Interviewer: ……………………………………………
